# Cryo‐electron microscopy of cholinesterases, present and future

**DOI:** 10.1111/jnc.15245

**Published:** 2020-12-10

**Authors:** Miguel Ricardo Leung, Tzviya Zeev‐Ben‐Mordehai

**Affiliations:** ^1^ Cryo‐Electron Microscopy Bijvoet Center for Biomolecular Research Utrecht University Utrecht The Netherlands; ^2^ The Division of Structural Biology Wellcome Centre for Human Genetics The University of Oxford Oxford UK

**Keywords:** cholinesterase, cryo‐electron microscopy, cryo‐electron tomography

## Abstract

Acetylcholinesterase (AChE) and butyrylcholinesterase (BChE) exist in a variety of oligomeric forms, each with defined cellular and subcellular distributions. Although crystal structures of AChE and BChE have been available for many years, structures of the physiologically relevant ChE tetramer were only recently solved by cryo‐electron microscopy (cryo‐EM) single‐particle analysis. Here, we briefly review how these structures contribute to our understanding of cholinesterase oligomerization, highlighting the advantages of using cryo‐EM to resolve structures of protein assemblies that cannot be expressed recombinantly. We argue that the next frontier in cholinesterase structural biology is to image membrane‐anchored ChE oligomers directly in their native environment—the cell.

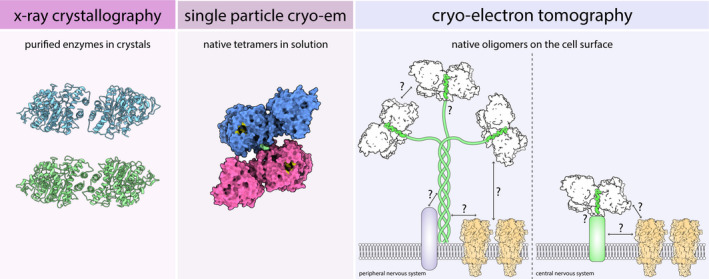

## Graphical abstract

The cholinesterases—acetylcholinesterase and butyrylcholinesterase—exist in the body primarily as oligomers. Although decades of work from many groups yielded numerous crystal structures of the cholinesterases, none of these structures captured the enzymes in their native oligomeric state. Recently, cryo‐electron microscopy was used to solve the structure of fully glycosylated human butyrylcholinesterase, revealing a non‐planar dimer of dimers stabilized by a superhelical core. In this brief review, we discuss how established and emerging cryo‐electron microscopy methods are poised to usher in the next era of cholinesterase structural biology.

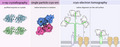

## 1 INTRODUCTION

The cholinesterases, acetylcholinesterase (AChE, E.C. 3.1.1.7) and butyrylcholinesterase (BChE, E.C. 3.1.1.8), are carboxylesterases specialized for hydrolyzing choline esters. AChE is present primarily in the nervous system, where it serves to terminate transmission at cholinergic synapses (Taylor, 1985). BChE is primarily present in plasma, where it is thought to act as a detoxifier (Johnson & Moore, 2012) and possibly to modulate ghrelin signaling (Chen et al., 2015). Crystal structures of AChE (Sussman et al., 1991) and BChE (Nicolet et al., 2003) have been available for many years. However, these structures captured cholinesterases (ChEs) primarily in dimeric forms, even though ChEs exist natively as tetramers or higher‐order oligomers (Legay, 2000).

Cholinesterase tetramers form through the interactions of the C‐terminal tryptophan amphiphilic tetramerization (WAT) helices of ChEs with a central proline‐rich peptide that runs antiparallel to the WAT helices (Boyko et al., 2019; Dvir et al., 2004; Leung et al., 2018). Because the proline‐rich peptide is contributed by a distinct gene product, the tetramer is, strictly speaking, a heteropentamer. However, for consistency with the literature, we refer to the heteropentameric assembly of four ChEs and one proline‐rich peptide as the “cholinesterase tetramer.”

Acetylcholinesterase tetramers are primarily anchored to the cell surface or the extracellular matrix. In the brain, AChE tetramers assemble around a proline‐rich peptide contributed by a transmembrane protein called the proline‐rich membrane anchor (PRiMA) (Perrier et al., 2002). At neuromuscular junctions, up to three AChE tetramers assemble around collagen Q (ColQ) (Krejci et al., 1997), which anchors the enzymes to the basal lamina (Hall, 1973; McMahan et al., 1978). In contrast, BChE exists primarily as a soluble tetramer, with the polyproline peptide derived from a variety of proteins. Most of the polyproline peptides in serum BChE are from lamellipodin (Li et al., 2008), whereas BChE recombinantly expressed in Chinese hamster ovary (CHO) cells can collect proline‐rich peptides from ~60 different proteins (Schopfer & Lockridge, 2016).

**Figure 1:**
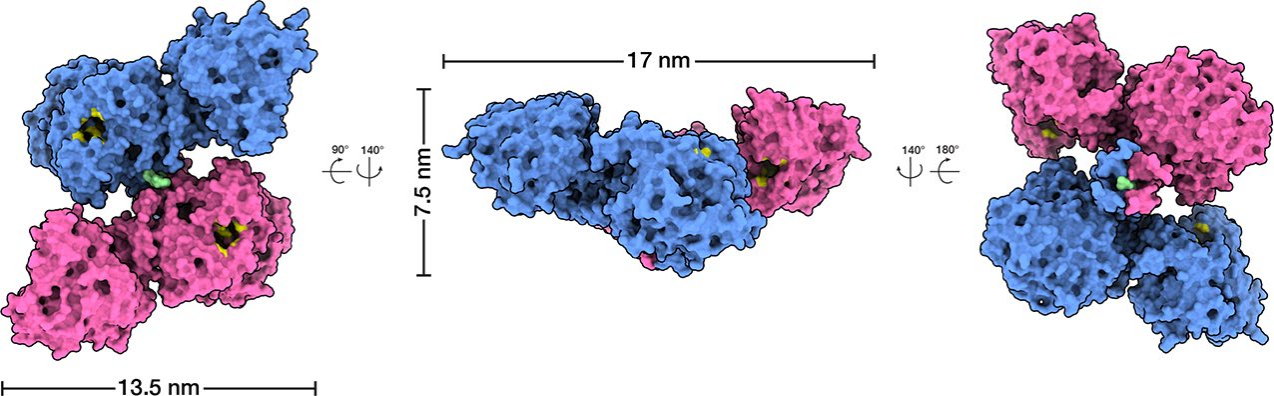
The native butyrylcholinesterase tetramer is a dimer of dimers assembled around a superhelical core. Molecular surface representations of the human butyrylcholinestrase tetramer made up of two canonical dimers (blue and pink) whose C‐terminal tryptophan amphiphilic tetramerization (WAT) helices assemble around a proline‐rich peptide (green). Residues of the catalytic triad (Ser198, Gly325, His438) are painted in yellow. The molecular surface was generated from PDB ID 6I2T, which was fit into the cryo‐EM map EMD‐4400

Although large quantities of tetrameric human BChE (HuBChE) can be purified from plasma (Lockridge et al., 2005), there have been no successful attempts to crystallize native tetrameric HuBChE despite many years of effort (Nachon et al., 2002). Recently, two groups independently used cryo‐electron microscopy (cryo‐EM) single‐particle analysis (SPA) to determine the structure of full‐length, fully glycosylated human BChE (HuBChE) tetramers purified directly from plasma (Boyko et al., 2019; Leung et al., 2018). These structures revealed that the BChE tetramer consists of a non‐planar dimer‐of‐dimers arranged around a central superhelical assembly that is largely shielded by the four catalytic domains (Figure 1). Because the superhelical assembly is the major interface among protomers, the fact that it is shielded by the catalytic domains may contribute to the exceptional stability of the BChE tetramer (Boyko et al., 2019; Leung et al., 2018). The non‐planar arrangement resolved in cryo‐EM structures differs appreciably from low‐resolution crystal structures and computational models of ChE tetramers (Bourne et al., 1999; Pan et al., 2009; Raves et al., 1998).

In this brief review, we reflect on how the case of the BChE tetramer illustrates how cryo‐EM SPA can help resolve structures of protein complexes that cannot be expressed recombinantly. Looking towards the future of ChE structural biology, we discuss how a related method, cryo‐electron tomography, enables structural studies in an even more native environment—that of the intact cell. We highlight questions of ChE organization that are ripe for exploration with new and emerging EM methods.

## 2 CRYO‐ELECTRON MICROSCOPY EXPANDS THE RANGE OF SAMPLES AMENABLE TO STRUCTURAL ANALYSIS

Why was it necessary to use native butyrylcholinesterase for structural studies? The answer is simple—recombinant human BChE (rHuBChE) does not efficiently form tetramers. Only 10%–30% of rHuBChE produced in cultured CHO cells is tetrameric (Blong et al., 1997; Saxena et al., 1998). Similarly, rHuBChE expressed in the milk of transgenic goats is primarily dimeric (Huang et al., 2007). Recombinant HuBChE produced by either approach is also incompletely glycosylated (Huang et al., 2007; Saxena et al., 1998), which affects the assembly and resulting stability of the tetramer. Although the population of tetramers can be increased by co‐expressing HuBChE with PRAD‐containing peptides in CHO cells, the resulting tetramers still have shorter circulatory half‐lives than native HuBChE (Duysen et al., 2002).

The properties that make native HuBChE exceptionally stable in circulation also made it exceptionally difficult to crystallize (Nachon et al., 2002). In particular, glycans account for nearly 25% of the molecular weight of HuBChE (Lockridge et al., 1987), but glycans introduce both compositional and conformational heterogeneity that can impede crystallization (Chang et al., 2007). Strategies to crystallize heavily glycosylated proteins include reducing glycan heterogeneity by expressing recombinant proteins in glycosyltransferase‐deficient cell lines, trimming glycans after expression through enzymatic deglycosylation, or removing glycosylation sites altogether through construct engineering (Chang et al., 2007; Lee et al., 2015). However, these approaches are clearly unsuitable when glycosylation is necessary for protein folding, stability, function, or complex formation.

Cryo‐EM SPA offers an alternative method to determine high‐resolution structures of macromolecules. The technique involves imaging individual molecules embedded in thin ice without the need of first forming highly ordered arrays, thus circumventing one of the stumbling blocks of crystallography. For reviews on the method see (Nogales & Scheres, 2015; Vinothkumar & Henderson, 2016). Indeed, SPA was used to solve structures of the heavily glycosylated HIV‐1 envelope glycoprotein, whose mass is almost ~50% glycan chains (Lee et al., 2015, 2016; Lyumkis et al., 2013). The field is advancing fast—resolution records are often short‐lived and labs around the world are constantly pushing the limits of the method. For recent reviews on cutting‐edge advancements see (Danev et al., 2019; Lyumkis, 2019; Murata & Wolf, 2018; Wu & Lander, 2020).

Of particular relevance to the cholinesterase field, SPA can deal with native or wild‐type material with little to no need for the extensive mutations and truncations that are often necessary to coax proteins to form crystals. That being said, SPA has its own challenges; perhaps the most pronounced is the need to get intact protein molecules randomly distributed in thin ice. Unfortunately, this is rarely the case on the first try. There is often a lot of screening and optimization that goes into preparing samples that can yield high‐resolution SPA structures (see Carragher et al., 2019 and Drulyte et al., 2018).

Nonetheless, SPA has been used in many cases to image protein material purified directly from tissue. Notable examples include structures of the ribosome‐bound Sec61 channel purified from pancreas (Voorhees et al., 2014), tau filaments purified directly from deceased brain tissue (Fitzpatrick et al., 2017), uromodulin filaments purified from human urine (Stanisich et al., 2020; Stsiapanava et al., 2020), and connexin channels purified from lens tissue (Flores et al., 2020). Because SPA requires relatively little material, an even more exciting prospect is to use the method as part of a structural proteomics pipeline in which protein complexes are fractionated and enriched directly from cell lysates, then analyzed in parallel with SPA and mass spectrometry (Ho et al., 2020; Kastritis et al., 2017; Verbeke et al., 2018). Information from these two methods can then be integrated and used by specialized software to essentially sequence by structure in cases where resolutions are better than about 4 Å (Ho et al., 2020).

## 3 WHAT'S NEXT FOR THE STRUCTURAL BIOLOGY OF THE CHOLINESTERASES?

**Figure 2:**
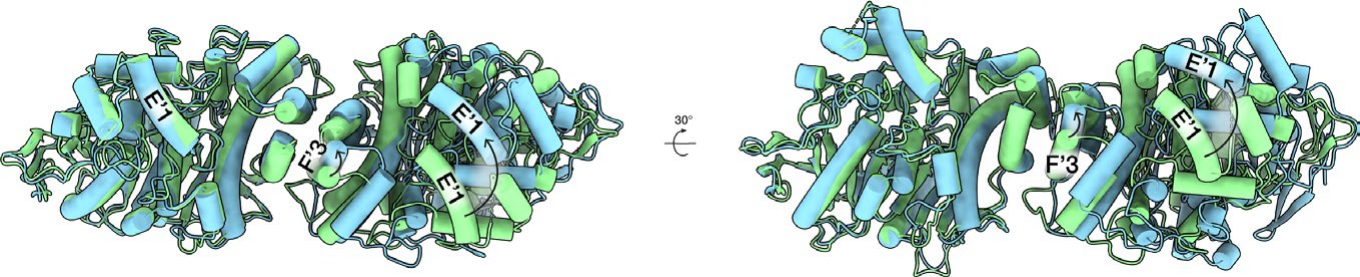
Cryo‐EM suggests fundamental differences between butyrylcholinesterase and acetylcholinesterase dimers. Catalytic domains of the human butyrylcholinesterase dimer (from PDB ID 6I2T, based on EMD‐4400) are shown in blue, whereas those from human acetylcholinesterase (from PDB ID 3LII) are shown in green

One clear follow‐up would be to attempt to resolve the structure of the native AChE tetramer. Fitting two BChE crystal structures into the cryo‐EM map resulted in a dimer that differed noticeably from the canonical AChE dimer (Figure 2) (Leung et al., 2018). Specifically, the structures differ in the relative orientations of the helices within the four‐helix bundle at the canonical dimer interface. If this difference is not caused by AChE truncation, then the AChE tetramer may very well be more planar than the BChE tetramer, as suggested by low‐resolution crystal structures of *Electrophorus electricus* AChE tetramers (Bourne et al., 1999; Raves et al., 1998). However, determining the structure of tetrameric AChE may not be as straightforward as it was for BChE. Although soluble tetrameric AChE can be prepared through trypsin digestion (Toker et al., 2020), it remains unclear whether such preparations will be suitable for SPA.

We argue that the next frontier in cholinesterase structural biology is to image AChE in the most native possible environment—the cell surface. While visualizing proteins in the cellular environment is infinitely harder than studying purified proteins, the methods needed to achieve this goal are maturing quite rapidly. Cryo‐electron tomography (cryo‐ET) leverages the uniquely multiscale capabilities of electron microscopy and combines this with the best possible structural preservation, allowing it to capture complete snapshots of the cellular environment at molecular resolution (Baker et al., 2017; Ng & Gan, 2020). In cryo‐ET, the sample is tilted relative to the electron beam and a series of projection images (called a tilt series) is acquired at discrete tilt increments over a defined angular range. Images in the tilt series are then aligned and back‐projected to yield a three‐dimensional reconstruction of the specimen. Cryo‐ET imaging is particularly challenging because the electron dose the sample can tolerate, which is already limited for radiation‐sensitive biological material (Glaeser, 1971), must be further spread out over a large number of images.

When it comes to cellular samples, one of the biggest hurdles is sample thickness. Although cells smaller than ~10 µm can be vitrified by plunge‐freezing in a manner similar to purified proteins, larger cells need to be frozen under high pressure in order to be uniformly vitrified throughout (Dubochet, 1995). Furthermore, most eukaryotic cells are too thick for high‐resolution imaging with a transmission electron microscope and must first be thinned prior to collecting tilt series. In a method called cryo‐electron microscopy of vitreous sections (CEMOVIS), blocks of frozen samples are physically sectioned with a cryo‐ultramicrotome and the sections transferred to cryo‐EM grids for imaging (Al‐Amoudi et al., 2004). In an alternative method, called cryo‐focused ion beam (cryo‐FIB) milling, a beam of ions is used to ablate material directly from cells grown on grids, leaving a thin lamella that can be directly imaged by cryo‐ET (Marko et al., 2007; Rigort et al., 2012). Localizing molecules of interest in the crowded environment of the cell is another major challenge for cellular cryo‐ET. If target molecules can be labeled fluorescently, they can be at least roughly localized by correlating cryo‐FIB milling and cryo‐ET with cryo‐fluorescence microscopy (called correlative light and electron microscopy, CLEM) (Gorelick et al., 2019; Moser et al., 2019; Wu et al., 2020).

When the target is present in multiple copies, these can be computationally extracted, aligned to a common reference, and averaged (Förster et al., 2005; Grünewald et al., 2003). This process, known as subtomogram averaging (reviewed in Leigh et al., 2019), serves to improve signal‐to‐noise ratio and increase resolution for the molecule/s of interest. The resulting averages can also be plotted back into their original particle positions and orientations, revealing spatial relationships between different molecular species (Albert et al., 2017; Levitan et al., 2019; Paul et al., 2020) or between different conformational states of a single complex (Asano et al., 2015; Lin & Nicastro, 2018).

Beyond the goal of studying cholinesterase structure, there are a number of questions that can only be addressed by an in situ structural biology approach. For instance, AChE’s function in terminating neurotransmission hinges on its precise localization to and concentration at the basal lamina of the neuromuscular junction. However, AChE organization at the neuromuscular junction involves more than just a binary interaction between AChE and ColQ (Karmouch et al., 2013; Massoulié & Millard, 2009). Heparin‐binding domains in ColQ interact with the heparan sulfate proteoglycan perlecan, which in turn interacts with the transmembrane protein dystroglycan (Peng et al., 1999), and the C‐terminus of ColQ also interacts with muscle‐specific kinase (MuSK) (Cartaud et al., 2004). Perlecan is expressed along the myofiber, but MuSK localizes specifically to the neuromuscular junction and therefore dictates the localization of ColQ‐tethered AChE (Cartaud et al., 2004). How is this membrane‐spanning multi‐protein complex arranged? How is ColQ‐tethered AChE spatially organized in the basal lamina and how does this relate to the distribution of MuSK in the cell membrane?

Acetylcholine receptors (AChRs), on the other hand, have been extensively studied with cryo‐EM. SPA was recently applied to determine the structure of the native receptor purified directly from the *Torpedo* electric organ and reconstituted in lipid nanodiscs (Mahfuzur Rahman et al., 2020). AChRs are known to form dense, ordered clusters on postsynaptic membranes (Cartaud et al., 2000) and cryo‐ET and subtomogram averaging have been applied to study these clusters (Zuber & Unwin, 2013). This study showed that individual receptors are bridged by up to three copies of the cytoplasmic scaffolding protein rapsyn. When rapsyn is absent, AChRs fail to cluster (Gautam et al., 1995). AChR clustering is also regulated by interactions with dystroglycan (Jacobson et al., 2001) and by MuSK‐mediated phosphorylation of receptor subunits (Lee et al., 2008; Mazhar & Herbst, 2012). Given the overlap between the molecular modules involved in AChR clustering and AChE anchoring, an interesting question is if and how might the distribution of AChE relate to the molecular organization of AChRs? What are the spatial relationships between AChE and AChR, both laterally in the membrane plane and in terms of distance from the cell surface?

How AChE is arranged in the central nervous system, where it is anchored by PRiMA, is much more poorly understood. Furthermore, AChE tetramers associate with ColQ differently than how they associate with PRiMA (Noureddine et al., 2008). In ColQ‐anchored AChE, monomers of one canonical dimer are linked by a disulfide bridge, whereas the other two monomers are each disulfide‐bonded to one of two cysteines flanking the ColQ PRAD. In contrast, all four AChE monomers can form disulfide links to the four cysteines upstream of the PRiMA PRAD (Noureddine et al., 2008). Comparing the architecture of ColQ‐tethered AChE with PRiMA‐anchored AChE would reveal whether these differences have large structural consequences on the relative arrangements between AChE tetramers and their anchors.

How might we visualize natively anchored forms of acetylcholinesterase? A key step will be choosing an appropriate platform. We could start by imaging minimal membrane systems, such as membrane vesicles purified from the electric organs of *Electrophorus* (Karlin, 1965) or *Torpedo* (Cartaud et al., 2000; Reed et al., 1975). As discussed earlier, such membrane preparations have been used to study the organization of AChR in native membranes (Unwin, 2017, 2020; Zuber & Unwin, 2013). AChE‐rich membrane fragments can be separated from AChR‐rich membranes through sucrose gradient centrifugation (Reed et al., 1975). Likewise, basal lamina ghosts containing large amounts of AChE can prepared by denervation and subsequent detergent extraction of frog muscle (Nicolet et al., 1986). These ex situ systems will facilitate high‐resolution imaging and subtomogram averaging, but they cannot be used to probe larger‐scale questions of how AChE interacts with other proteins of the membrane or the basal lamina.

Co‐expressing AChE with ColQ or with PRiMA in standard laboratory cell lines used for protein production could also result in cell surface‐anchored tetramers. A more physiologically relevant initial target, however, would be cultured neurons or myocytes, either primary cultures or immortalized cell lines (Thullbery et al., 2005). Imaging studies reveal that PRiMA‐anchored AChE forms discrete clusters along nerve fibers of cultured neurons (Rotundo & Carbonetto, 1987; Xie et al., 2010). These clusters could be suitable targets for in situ cryo‐ET, although a CLEM‐based targeting approach, for instance using fluorescently labeled fasciculin (Krejci et al., 2006), will likely be necessary.

Targeting the neuromuscular junction will be more difficult, but cell culture models do exist (Jevsek et al., 2004; Mis et al., 2005, 2017; Vilmont et al., 2016). Because these culture systems are extremely complex, initial experiments to determine the feasibility of this approach are necessary, for instance to evaluate whether neuromuscular junctions even form and mature on EM grids. Another approach would be to use neuromuscular organoids (Faustino Martins et al., 2020) or tissue harvested from surgical procedures (Jones et al., 2017), but preparing such samples for cryo‐ET will require an extremely challenging pipeline involving high‐pressure freezing and cryo‐FIB/lift‐out, as described in (Schaffer et al., 2019). While visualizing cell surface‐anchored AChE in the native context of a neuromuscular junction may be a lofty goal, such studies would have far‐reaching implications beyond the immediate cholinesterase field.

## 4 CONCLUDING REMARKS

Decades of X‐ray crystallography studies on ChEs unveiled the chemical wonders of their very high catalytic rates. Likewise, these studies were instrumental in defining the modes of action of AChE inhibitors, thus facilitating rational drug design. Cryo‐EM SPA now extends our knowledge to the native quaternary structure of BChE, solving a long‐standing question in the field. Looking to the horizon of ChE structural biology, we hope cryo‐ET will enable us to image ChE oligomers and their interacting partners directly in their native environments, uniting the molecular and cellular scales and bringing the ChE back into its functional context.

## CONFLICT OF INTEREST

We declare no competing interests.

## AUTHOR CONTRIBUTIONS

MRL and TZ wrote the manuscript.
